# Research into and Application of a Flexible Piezoelectric Stacked Ultrasonic Sensor Based on ZnO/PVDF-Modified Materials

**DOI:** 10.3390/nano16161045

**Published:** 2026-08-21

**Authors:** Wei Liu, Yunlai Shi, Zhijun Sun, Yuanyuan Wang

**Affiliations:** State Key Laboratory of Mechanics and Control of Mechanical Structures, Nanjing University of Aeronautics and Astronautics, Nanjing 210016, China

**Keywords:** P(VDF-TrFE), ZnO, flexible piezoelectric sensor, ultrasonic, thickness measurement

## Abstract

As the primary carrier for oil and gas transportation, pipelines are critical for the entire industry. Pipelines are continuously subjected to corrosion and abrasion in the oil and gas delivery process, leading to gradual wall thickness reduction, shortened service life, and deteriorated operational safety. Ultrasonic testing has been widely adopted for monitoring pipeline wall thickness. Conventional ultrasonic transducers possess rigid configurations, which hinder large-area inspection and exhibit poor adaptability to complex curved components. In contrast, flexible ultrasonic sensors show prominent advantages, with their small size, light weight, and excellent conformal contact with curved surfaces. Flexible piezoelectric thin-film sensors have been used in a wide range of fields. As one of the most representative piezoelectric polymers, poly(vinylidene fluoride–trifluoroethylene) (P(VDF-TrFE)) combines favorable piezoelectric coefficients and intrinsic flexibility, making it popular. Some research groups have investigated the influences of modified filler particles, doping ratios, and fabrication process optimization on the performance of P(VDF-TrFE)-based piezoelectric composites, while others have concentrated on the practical applications of existing flexible piezoelectric sensors. This study emphasizes a rapid customized fabrication strategy for flexible sensors instead of single-specification standardized probes; hence, it does not share the same comparison benchmark as conventional fixed-dimension sensors. Systematic research on flexible piezoelectric thin-film sensors is presented, including piezoelectric material modification, substrate design, laminated structural design, fabrication workflows, establishment of the testing platform, and the development of matched circuit systems. The material preparation and manufacturing processes are optimized, and a scalable technical route for fabricating flexible piezoelectric sensors is proposed. Using this route, flexible piezoelectric thin-film sensors can be rapidly tailored for different application scenarios to satisfy diverse engineering demands. Multiple experiments were conducted on pipeline samples with varying wall thicknesses and curvatures. The results verify that the sensor reaches a measurement precision of 0.01 mm, meeting the demands of high-precision pipeline structural health monitoring.

## 1. Introduction

At present, pipelines serve as one of the primary modes of transport in industries such as petrochemicals and natural gas, and the health of a pipeline directly impacts production safety and economic efficiency. As the service time increases, pipeline walls gradually thin due to corrosion, erosion, wear, aging, and other factors, potentially leading to fractures, severe safety incidents, and economic losses. Therefore, monitoring the pipeline wall thickness is crucial, as it helps to promptly identify potential safety hazards and provides a reference for assessing the pipeline’s lifespan, ensuring production safety and continuity [1].

Traditional ultrasonic thickness sensors are hard and rigid structures, with many limitations in practical applications. On the one hand, ultrasonic sensors made of hard piezoelectric ceramic material (PZT) and do not fit well against the curved surface of the pipe wall, resulting in only line or point contact. There is high energy loss of the ultrasonic sensor during the process of entering the pipe wall, which affects the amplitude and signal-to-noise ratio of the detection signal, which then affects the measurement accuracy of the system [2]. On the other hand, due to the large volume and weight of traditional sensors, it can be difficult to install them at important detection points with limited space.

**Figure 1:**
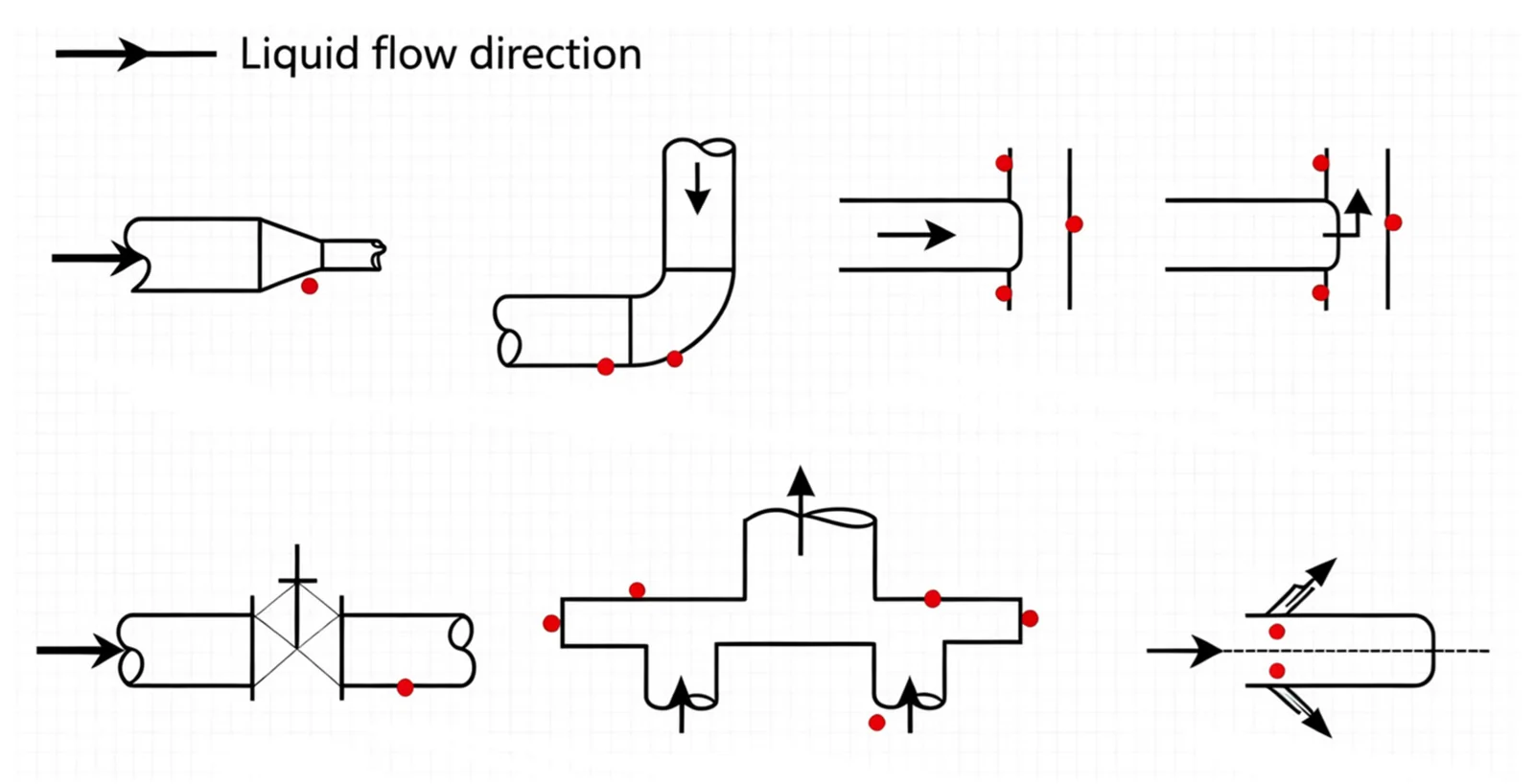
The red markers indicate the most critical test points of the pipeline.

The normal detection points are shown in Figure 1, mostly at locations of variable diameter, bent pipes, and blind heads. These locations are often characterized by complex stress conditions and limited space, in which case flexible ultrasonic sensors can adapt to more complex pipeline shapes and installation spaces. Flexible ultrasonic sensors also have low density and a light weight, making it easier to implement an array-based sensor monitoring network, which has advantages in large-scale pipeline monitoring applications.

In 2021, Ibrahim AlMohimeed designed and prepared a double-layer PVDF wearable ultrasonic sensor. The optimized multilayer-stacked architecture increased the piezoelectric coupling coefficient, yielding a stronger ultrasonic transmitting and receiving response. It was successfully used for the in vivo measurement of skeletal muscle thickness and the evaluation of muscle contraction parameters [3]. In 2023, a research team from Graz University of Technology studied the fully printed manufacturing process of P(VDF-TrFE) flexible ultrasonic transducers, achieving precise control of a single-layer thickness of approximately 2 um, providing a foundation for understanding the correlation between manufacturing processes and thickness measurement performance [4]. The Zhang group systematically reviewed four categories of strategies for PVDF-based materials: molecular and chemical structure regulation, crystallization structure engineering, composite system and interface modulation, and multi-scale macroscopic structure design [5]. Their work utilized only pristine P(VDF-TrFE) films without ZnO or other filler modifications. In addition, the sensor featured a fixed array layout, which precluded rapid customized design for different practical scenarios. In 2025, W. Liu fabricated dual-structure synergistically reinforced MXene/P(VDF-TrFE) piezoelectric nanocomposites for dynamic pressure monitoring [6]. No ultrasonic thickness measurement was carried out, and MXene instead of ZnO was adopted as the filler in the piezoelectric material system. Li Yehai and Guo Shifeng systematically reviewed the lightweight conformal sensing network and showed that PVDF can be made into ultra-thin films due to its excellent flexibility, which is suitable for large-area curved surface monitoring and provides a framework for the positioning of PVDF in structural health monitoring [7]. O.V. Muraveva proposed a comprehensive evaluation method for wall thickness non-uniformity based on PVDF flexible piezoelectric transducers and small-diameter pipeline section acoustic propagation models, and verified its consistency through local ultrasonic thickness measurement [8].

Traditional flexible piezoelectric sensors may suffer in terms of performance and testing accuracy, due to relatively large errors, making them difficult to apply in practice [9]. In this article, the process optimization and doping modification of P(VDF-TrFE) piezoelectric materials are discussed. Here, the performance of the material was improved. Through theoretical simulation and analysis of the laminated design of flexible piezoelectric sensor, a flexible sensor was prepared independently. At the same time, flexible sensor performance test equipment and ultrasonic driven test equipment were designed to test the performance of the flexible sensor. Finally, experimental verification was carried out on the film and pipeline, which provides a reference for the development of health monitoring solutions. Through this research, more efficient and accurate long-term online monitoring of the pipeline wall thickness can be achieved, providing a reference for developing solutions for pipeline health monitoring.

## 2. Experimental Instruments and Materials

The materials used in this article are listed in Table 1, including the most basic P(VDF-TrFE) powder, zinc oxide (ZnO) nanoparticles incorporated as fillers into the P(VDF-TrFE) matrix, solvent butanone used to dissolve P(VDF-TrFE) powder, and an An–Ag target used for electrode deposition by magnetron sputtering.

**Figure 2:**
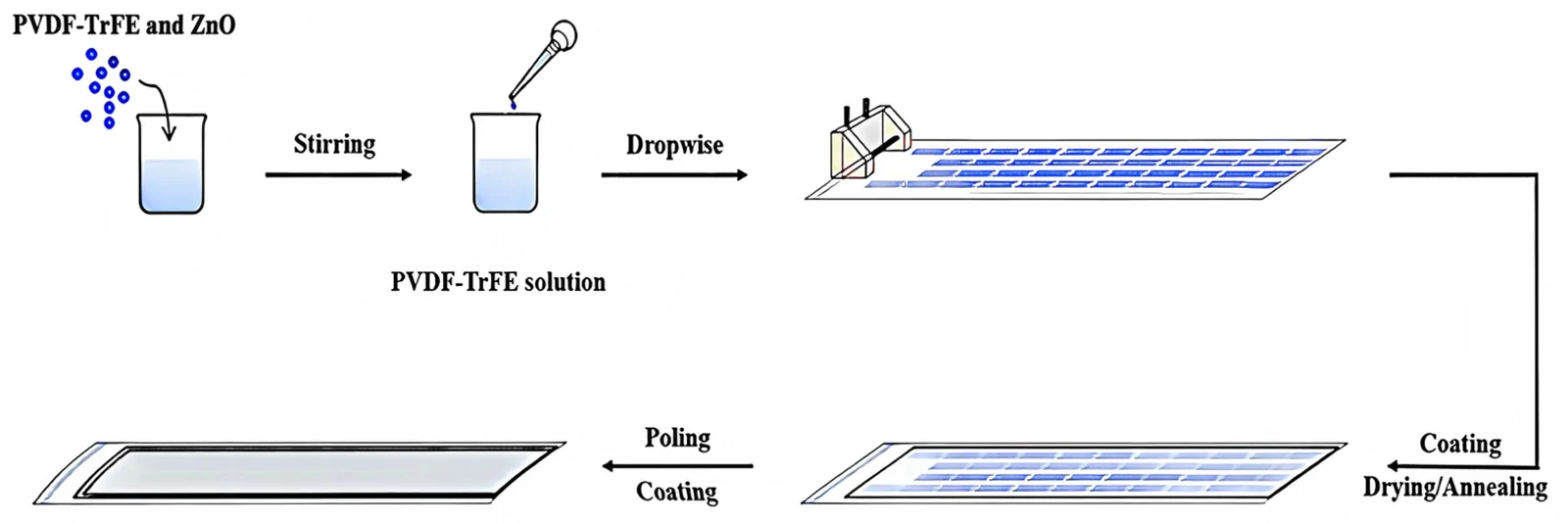
Preparation process of P(VDF-TrFE).

The preparation process of the P(VDF-TrFE)-based piezoelectric film is shown in Figure 2, including the solution, coating film, drying treatment, high-temperature annealing treatment, polarization, and other piezoelectric preparation processes.

The main components used to prepare the P(VDF-TrFE) films are listed in Table 2. First, we prepared the experimental equipment required for preparing the slurry, cleaned the experimental vessels, dried them in an oven, and set them aside. Then, we took 50 mL of butanone solution and placed it in a conical flask. We weighed 5.52 g of P(VDF-TrFE) powder and 5% ZnO particles using an electronic balance, then poured them together into a conical flask and stirred until the bubbles completely disappeared to complete the slurry preparation.

The next step was coating, which involves uniformly applying the paste onto the surface of the FPC circuit board. First, we fixed the cleaned FPC circuit board onto the glass board with adhesive tape, ensuring that the entire FPC was completely adhered to the surface of the glass board without gaps, in order to avoid an uneven film thickness caused by the copper foil bulging during the coating process. Then, we placed the glass plate on the coating machine and turned on the vacuum pump to fully adhere the glass plate to the coating machine to prevent it from sliding. Next, we adjusted the scale of the scraper to 440 um and placed it on the FPC on the glass plate. Then, we use a dropper to extract an appropriate amount of slurry and slowly and evenly applied it to the front of the blade for coating.

After coating was completed, we placed the FPC circuit board and glass substrate into a vacuum drying oven, closed the air outlet valve, and turned on the air compressor switch for vacuum drying, in order to fully evaporate the butanone and achieve a dry film. Annealing is a crucial step to increase the crystallization ratio of the β-phase after the formation of P(VDF-TrFE) thin films. We placed the above glass plate in a 140 °C oven and removed it after about 1 h. This process promotes the crystallization of thin films and improves their performance.

In the final step of polarization treatment, we deflected the dipole of the β-phase crystal in the P(VDF-TrFE) dry film towards the direction of the electric field under high voltage to form a uniformly strong polarization electric field between the upper and lower electrodes. After about 10 min, the FPC circuit board was removed to obtain a piezoelectric film with piezoelectric effect.

## 3. Optimization of the Preparation Process and Doping Modification of P(VDF-TrFE) Thin Films

Piezoelectric material is the basic sensitive unit of a piezoelectric ultrasonic sensor, and its performance parameters are significant for the sensor design [10]. P(VDF-TrFE)/ZnO composite films with various filler loadings were fabricated via solution blending and spin-coating. The ZnO doping content is defined as the weight percentage (wt%) of ZnO nanoparticles relative to the total mass of P(VDF-TrFE) solid powder [11]. The commercial ZnO nanoparticles used in this work exhibit uniform equiaxed morphology with an average particle size of 30–50 nm; no obvious preferred crystallographic orientation or nanorod structure was observed. To explore the impact of doping on material properties, we chose zinc oxide (ZnO) as the doping material and set multiple doping ratios (3, 5, 7, 9, and 11 wt%). We conducted research on the surface morphology, crystal structure, and piezoelectric properties of the thin films. Here, the mixture was magnetically stirred continuously at 60 °C for 4 h to obtain a homogeneous and transparent solution.

Subsequently, ZnO nanoparticles were gradually added into the P(VDF-TrFE) solution in batches to prevent particle agglomeration. The mixed suspension underwent mechanical stirring for 2 h, followed by ultrasonic dispersion at a power of 150 W for 30 min to improve the dispersion uniformity of the nanoparticles. After sufficient defoaming, the well-dispersed composite suspension was spin-coated onto clean flexible PI substrates at 3000 rpm for 40 s. The wet films were predried at 80 °C for 2 h to eliminate the residual solvent. After thermal annealing at 140 °C for 1 h, the P(VDF-TrFE) achieved a high fraction of β-phase crystals. Under a poling voltage of 7.5 kV, the ZnO-incorporated P(VDF-TrFE) film delivered a longitudinal piezoelectric coefficient up to 27 pC/N.

**Figure 3:**
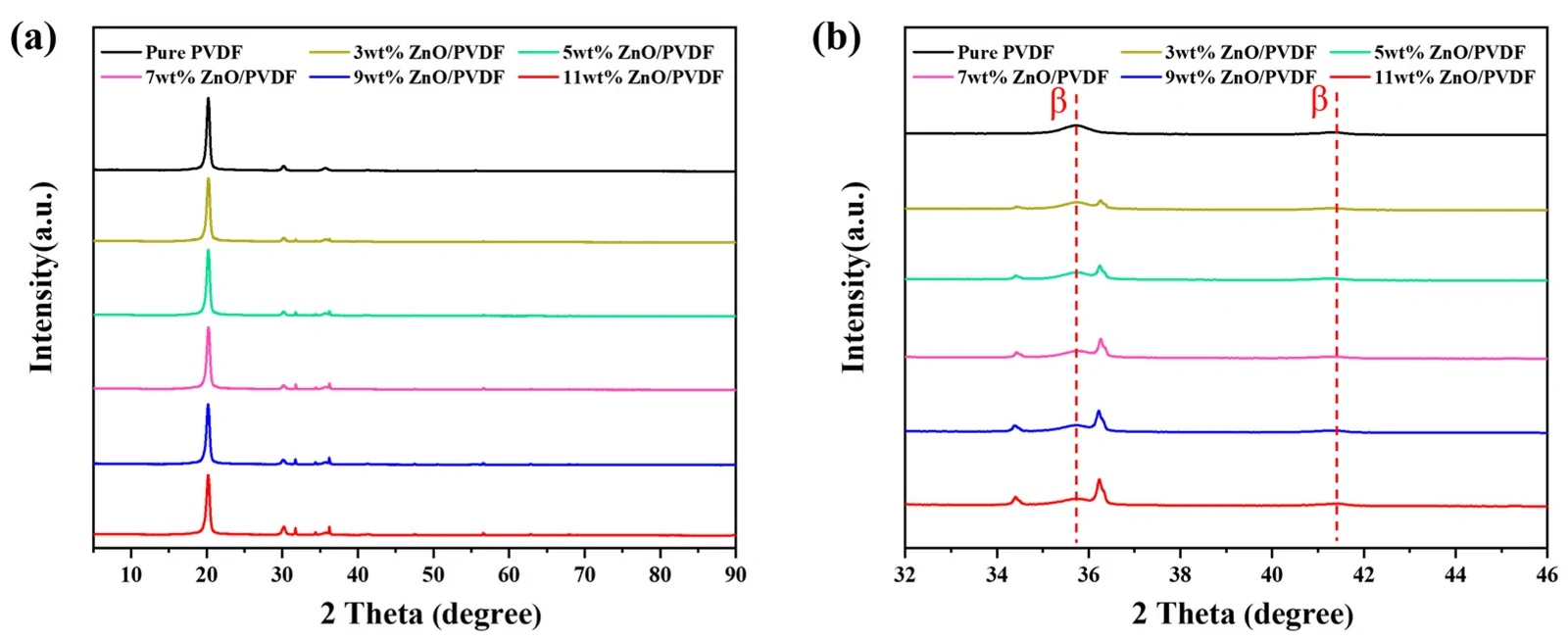
(**a**) ZnO/P(VDF-TrFE) XRD spectra, (**b**) Magnified view of the β-phase.

The effect of different ZnO doping amounts on the crystallization properties of ZnO/P(VDF-TrFE) composite copolymer was analyzed using XRD. In the XRD patterns, the peaks at 20°, 35.8° and 41.2° represent the (110)/(200) plane, (001) plane, and (111)/(201) plane of β-phase crystal [12,13]. Due to the pseudohexagonal unit cell of β-phase P(VDF-TrFE), the interplanar spacings of (110) and (200), as well as (111) and (201), were nearly equivalent, resulting in overlapping diffraction peaks that could not be separated in the present XRD patterns. This crystallographic feature was originally elucidated by Ohigashi for P(VDF-TrFE) copolymers. The diffraction peak near 20.0° corresponds to the overlapping (110)/(200) planes of the β-phase P(VDF-TrFE). The peak intensity declined after introducing ZnO nanoparticles. This tendency reveals that ZnO restricts the ordered packing of P(VDF-TrFE) molecular chains, which reduces the crystallinity and alters the relative β-phase content of the composite films. As shown in Figure 3b, the diffraction peak intensity at 35.8° rose markedly with increasing ZnO loading, while the peak intensity at 41.2° exhibited a modest enhancement. These findings suggest that the β-phase crystal content in the ZnO/P(VDF-TrFE) composite copolymer tends to increase with higher ZnO content.

The characteristic peaks at 31.8°, 34.4°, 36.3°, 47.5°, 56.6°, 62.9°, 66.4°, 67.9°, and 69.0° were the (100), (002), (101), (102), (110), (103), (200), (112), and (201) crystal plane diffraction peaks of ZnO, respectively. The peak type of the diffraction peak was sharp, indicating that the sample has good crystallinity, and the position of the corresponding crystal plane diffraction peak of ZnO was basically unchanged, indicating that the crystal form of ZnO in the ZnO/P(VDF-TrFE) composite copolymer did not change.

**Figure 4:**
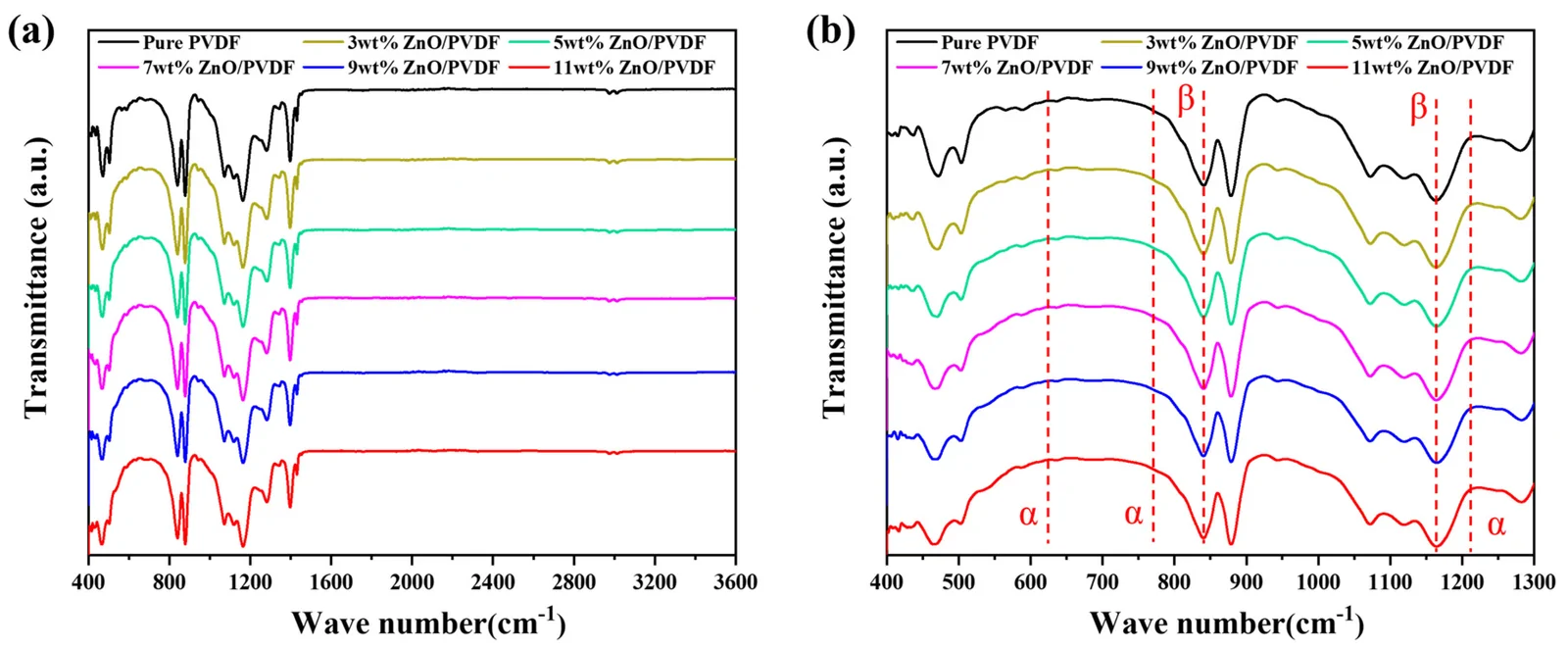
(**a**) ZnO/P(VDF-TrFE) FTIR spectra, (**b**) Magnified view of the spectra.

Figure 4 shows the FTIR spectra of ZnO/P(VDF-TrFE) composite copolymers with different ZnO doping amounts. According to previous reports, the absorption peaks of α-phase of P(VDF-TrFE) are at 614 cm^−1^, 764 cm^−1^, 976 cm^−1^ and 1234 cm^−1^, and the absorption peaks of its β-phase are at 470 cm^−1^, 510 cm^−1^, 840 cm^−1^ and 1073 cm^−1^.

At wavenumbers 470 cm^−1^, 510 cm^−1^, 840 cm^−1^, 1073 cm^−1^, and 1431 cm^−1^, the characteristic absorption peaks of CF2 bending, CF2 swinging, and CH vertical to the plane of the main chain of the piezoelectric β-phase of PVDF appeared, and at wavenumber 880 cm^−1^, the characteristic absorption peaks of CH vertical to the plane of the main chain of the non-piezoelectric α-phase of PVDF appeared, which indicated that the film was in a polycrystalline state. Hence, there was no chemical correlation between PVDF and ZnO. No new characteristic absorption bands were observed in the FTIR spectra, implying that no obvious chemical reaction occurs between the P(VDF-TrFE) matrix and ZnO nanoparticles within the detection limit of FTIR. The effect of ZnO on the piezoelectric properties of the composite film is a physical effect. As a conductive filler, ZnO can enhance the local electric field strength of PVDF film during electric field polarization, enhancing the residual polarization strength of PVDF [14].

Based on the Beer–Lambert law, the relative fractions of the β-phase, F(β), for pure P(VDF-TrFE) and ZnO/P(VDF-TrFE) composites with ZnO loadings of 3, 5, 7, 9, and 11 wt% were determined to be 20.51%, 20.72%, 23.49%, 22.03%, 24.81%, and 23.56%, respectively (Table 3). The elevated F(β) values upon ZnO incorporation suggest a promoted formation of the β-phase, in good agreement with the XRD analysis.

**Figure 5:**
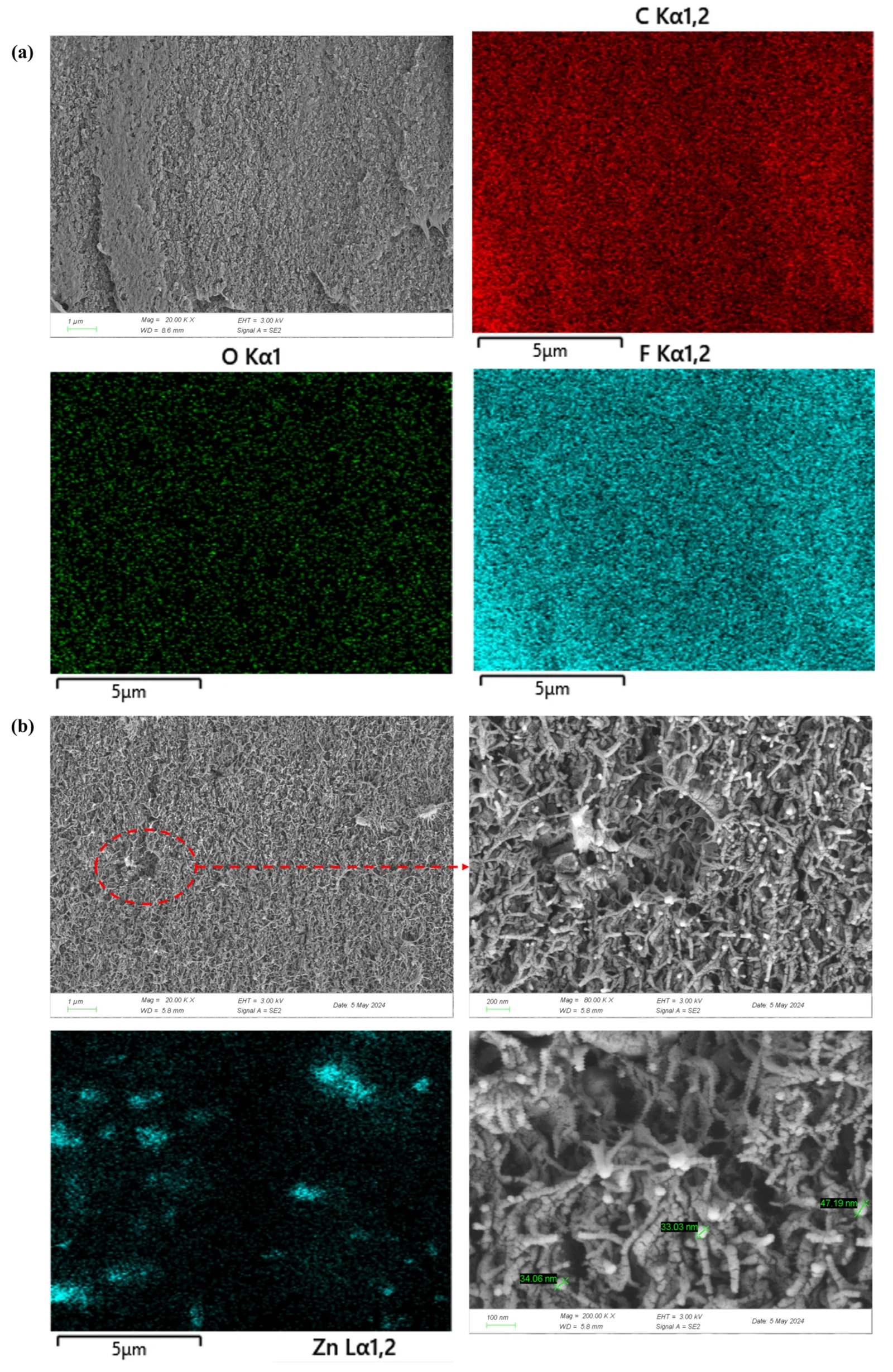
(**a**) SEM-EDS image of pure P(VDF-TrFE), (**b**) SEM-EDS image of ZnO/P(VDF-TrFE). The red dashed circle denotes the magnified view.

Figure 5 shows the SEM-EDS image of the ZnO/P(VDF-TrFE) composite copolymer. The surface microstructures of neat P(VDF-TrFE) and ZnO/P(VDF-TrFE) composite films were characterized using SEM. As shown in Figure 5a, the pure P(VDF-TrFE) film presents a relatively smooth and compact surface. After introducing ZnO nanoparticles, obvious differences in surface morphology emerge. High-magnification SEM images and corresponding SEM-EDS elemental mappings of Zn distribution reveal that, through the adopted fabrication process, the incorporated ZnO nanoparticles, with dimensions in the nanometer range, are uniformly dispersed throughout the P(VDF-TrFE) polymer matrix. At a low filler loading, ZnO nanoparticles are dispersed relatively uniformly within the polymer matrix, with limited agglomeration and tight interfacial contact between nanoparticles and P(VDF-TrFE). As the ZnO content increases, the probability of particle aggregation rises, and larger agglomerated domains appear on the film surface. Severe agglomeration creates visible interfacial gaps between the inorganic phase and polymer matrix, which may impair the load transfer effect. The incorporation of ZnO also disturbs the regular stacking of P(VDF-TrFE) molecular chains, altering the surface flatness and microstructure of the composite film.

**Figure 6:**
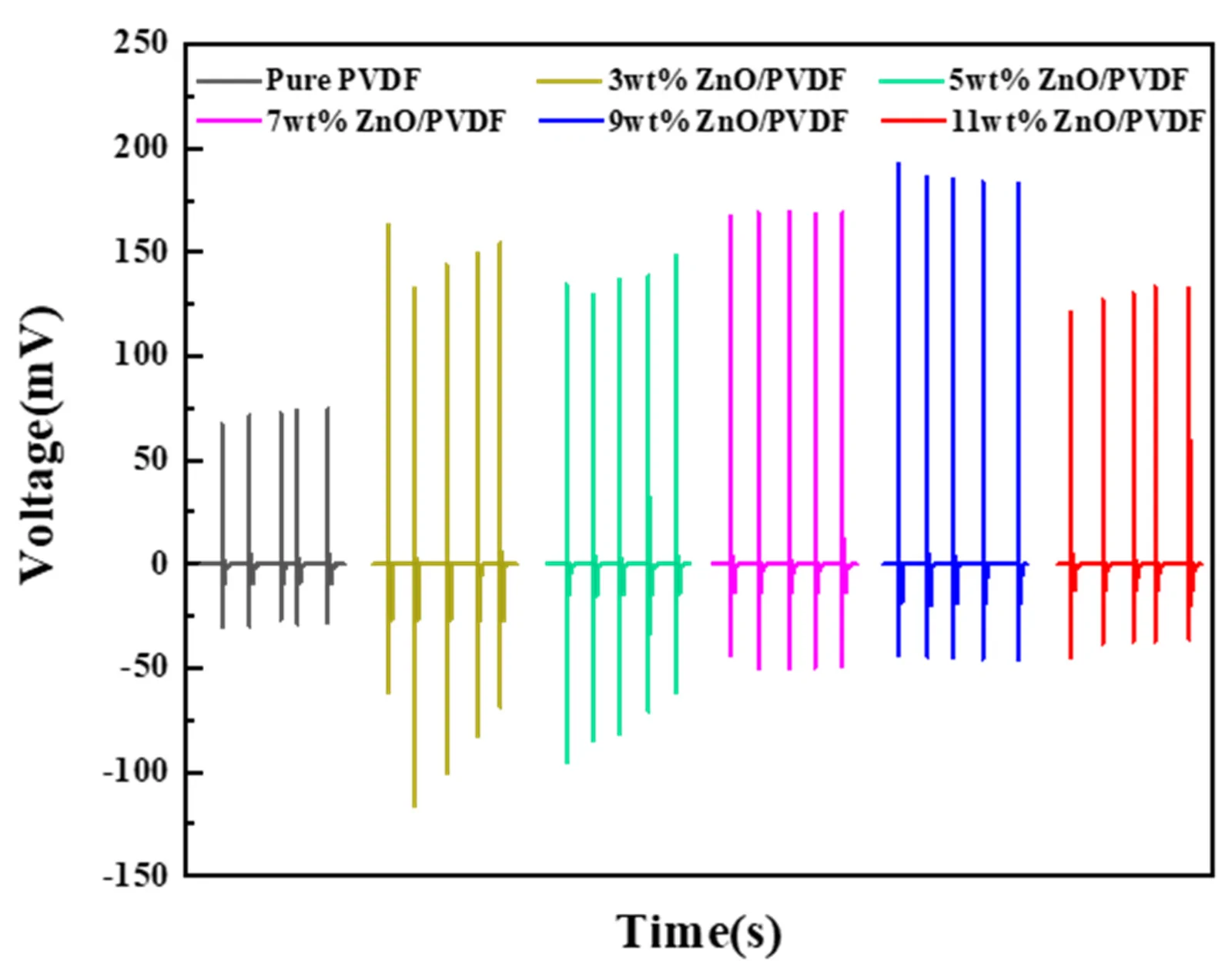
Doping ZnO with different ratios for piezoelectric properties and voltage values.

Figure 6 records the output electrical signals of ZnO/P(VDF-TrFE) composite copolymers with different ZnO doping levels. With the increase in the ZnO doping amount, the output voltage signal of the ZnO/P(VDF-TrFE) composite copolymer increases. When the doping amount of ZnO is 9 wt%, the output voltage signal of the ZnO/P(VDF-TrFE) composite material reaches the highest level. When the doping amount of ZnO is 11 wt%, the output voltage signal of the ZnO/P(VDF-TrFE) composite material decreases. It is possible that with the introduction of ZnO, the steric hindrance effect of C-F dipoles can increase, and excessive ZnO can lead to excessive steric hindrance, reducing the formation of β-phase crystals in the copolymer and thereby reducing the output voltage signal of ZnO/P(VDF-TrFE) composite materials.

## 4. Design and Ultrasonic Testing of Flexible Piezoelectric Sensors

In order to facilitate the design and processing of flexible thin-film piezoelectric sensors, a PI-based FPC circuit board was designed and corresponding FPC circuits were designed according to the required sensor size, test point location, and quantity requirements [15]. PI possesses favorable mechanical flexibility, high thermal stability, and excellent dielectric properties, making the FPC substrate compatible with flexible piezoelectric composite films.

### 4.1. Sensor Design and Fabrication

**Figure 7:**
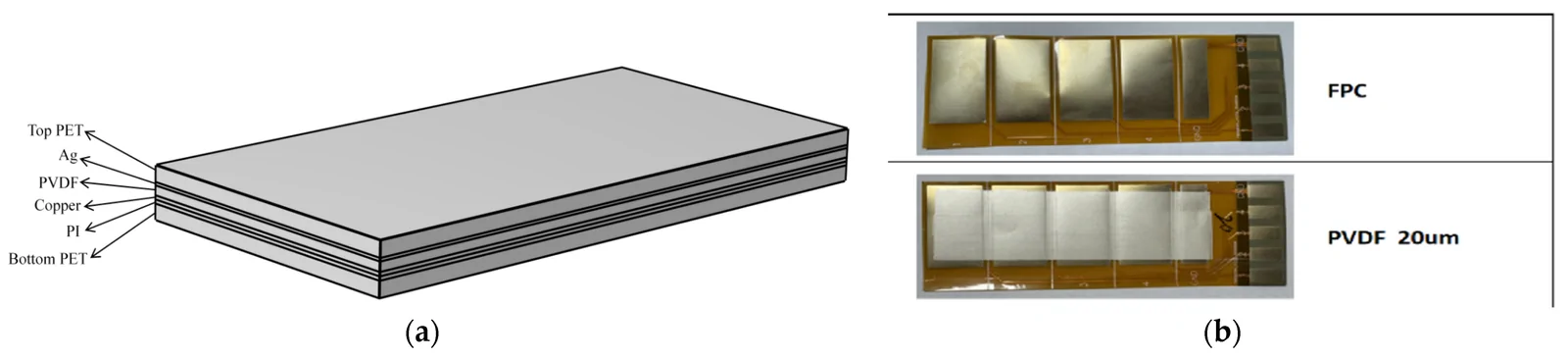
(**a**) Stacking design of flexible piezoelectric sensors; (**b**) piezoelectric thin-film sensor substrate.

The substrate design of this sensor adopts a laser-cut FPC circuit board [16]. There are four arrayed electrodes in the center of the flexible circuit board based on the PI substrate. Each electrode is connected to the gold finger area at the top for the export of electrical signals. The common metal electrode is above the film. The physical object of the whole sensor is shown in Figure 7.

**Figure 8:**
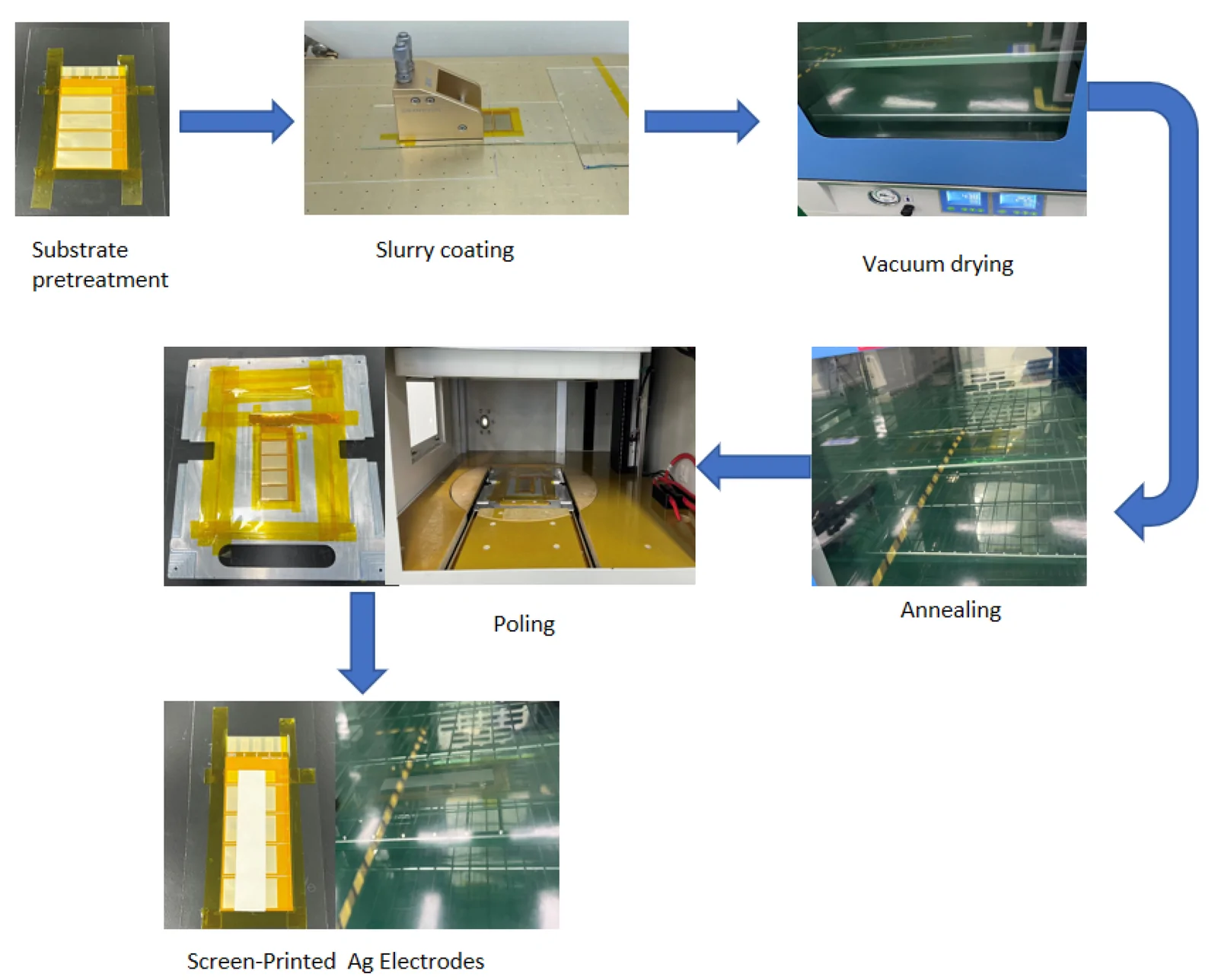
Manufacturing process of the pressure film sensor.

The manufacturing process of the PVDF flexible piezoelectric ultrasonic sensor is shown in Figure 8. First, lay the FPC substrate to be coated flat on a glass plate and fix its four edges with high-temperature tape. Prepare the slurry, and then adjust the doctor blade to the required height, i.e., the wet-film thickness. Place the coated substrate together with the glass substrate into a vacuum oven to dry the wet film into a dry film. Transfer the vacuum-dried dry film to a forced-air oven for annealing and crystallization. Put the annealed substrate along with the glass onto a dedicated poling tray and secure its four edges with high-temperature tape. Connect the tray to the substrate pads using copper foil to achieve electrical conduction. Lay the poled FPC substrate together with the glass flat on the tabletop, and perform screen-printing of the top electrode on the gold surface using the corresponding screen stencil. After printing, transfer the sample into a forced-air oven for drying.

The design of different sensor stack materials and stack thicknesses strongly affects the sensor performance parameters (sensor capacitance, resonant frequency, amplitude frequency characteristics, etc.) [17]. Following the above process flow, the sensor-related stack design parameters and basic piezoelectric performance parameters are listed below.

### 4.2. Ultrasonic Testing of Flexible Piezoelectric Sensors

PVDF piezoelectric film is a bidirectional passive device based on the piezoelectric effect, which can transmit and receive ultrasonic waves [18]. When the PVDF piezoelectric film is excited by the high-voltage pulse generated by the transmission circuit, it undergoes deformation due to the inverse piezoelectric effect, thereby emitting ultrasonic waves. After ultrasonic waves come into contact with the tested object, they reflect back and act on the PVDF piezoelectric film, causing it to vibrate under pressure and undergo stress deformation. A positive piezoelectric effect generates an electrical signal.

**Figure 9:**
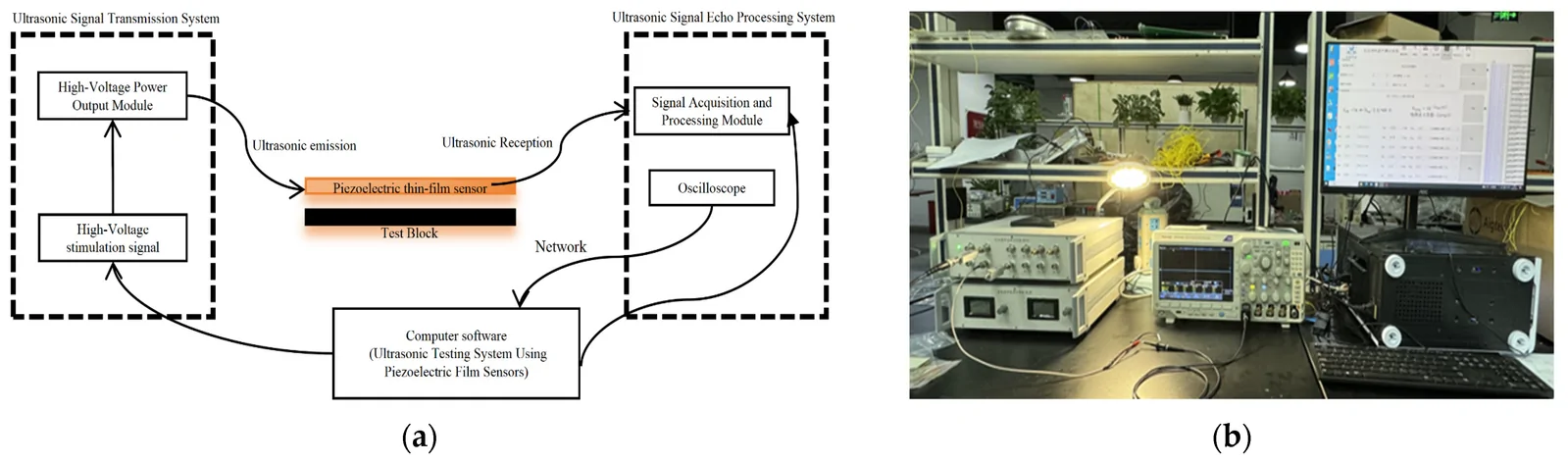
(**a**) Flexible piezoelectric sensor ultrasonic performance testing platform; (**b**) ultrasonic testing system.

This subsection examines the acoustic characteristics of flexible piezoelectric sensors. An ultrasonic detection system was built, which included an ultrasonic emission system and an ultrasonic echo signal system. The overall design block diagram of the system and practical experimental platform is shown in Figure 9.

The ultrasonic detection system included the hardware design of the ultrasonic transmission circuit, the receiving circuit, and computer configuration software, while using an oscilloscope to observe the waveform of ultrasound signals.

**Figure 10:**
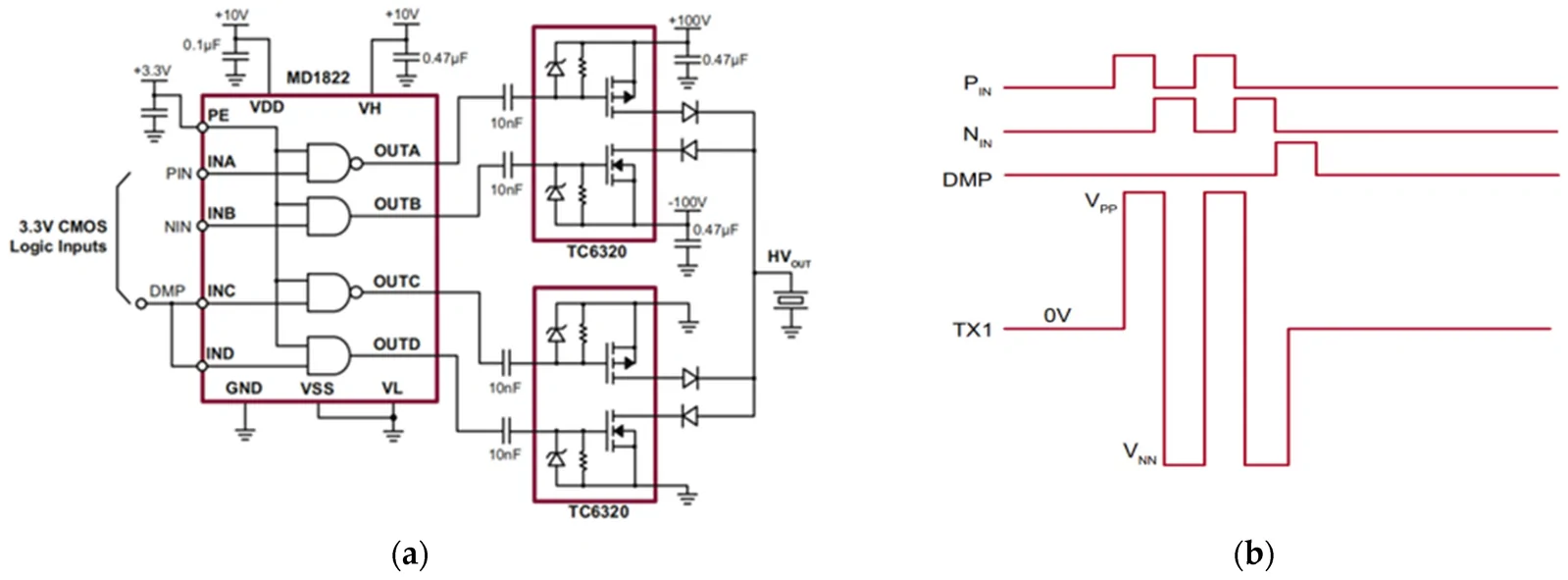
(**a**) Schematic of MD1822 and TC6320 circuits; (**b**) MD1822 control timing diagram.

The ultrasonic transmission circuit uses a frequency adjustable continuous pulse excitation method to excite piezoelectric materials with different resonant frequencies to generate echo signals. Figure 10 shows the circuit that mainly realizes switch control of the high-voltage power supply. And the circuit design was composed of an MD1822 MOSFET driver and TC6320 MOSFET. MD1822 is a high-speed 4-channel MOSFET driver, while TC6320 features a pair of high-speed and high-voltage complementary P and N-MOSFET, using FPGA programming to input MD1822 and output the corresponding TX square wave pulse signal from the chip. The frequency of the input timing determines the frequency of the output signal TX. The design parameters of this ultrasonic testing system were an output voltage of 0 V~+/−50 V and an excitation frequency of 1 MHZ~20 MHz, which were used to test the performance of the designed flexible piezoelectric sensor.

**Figure 11:**
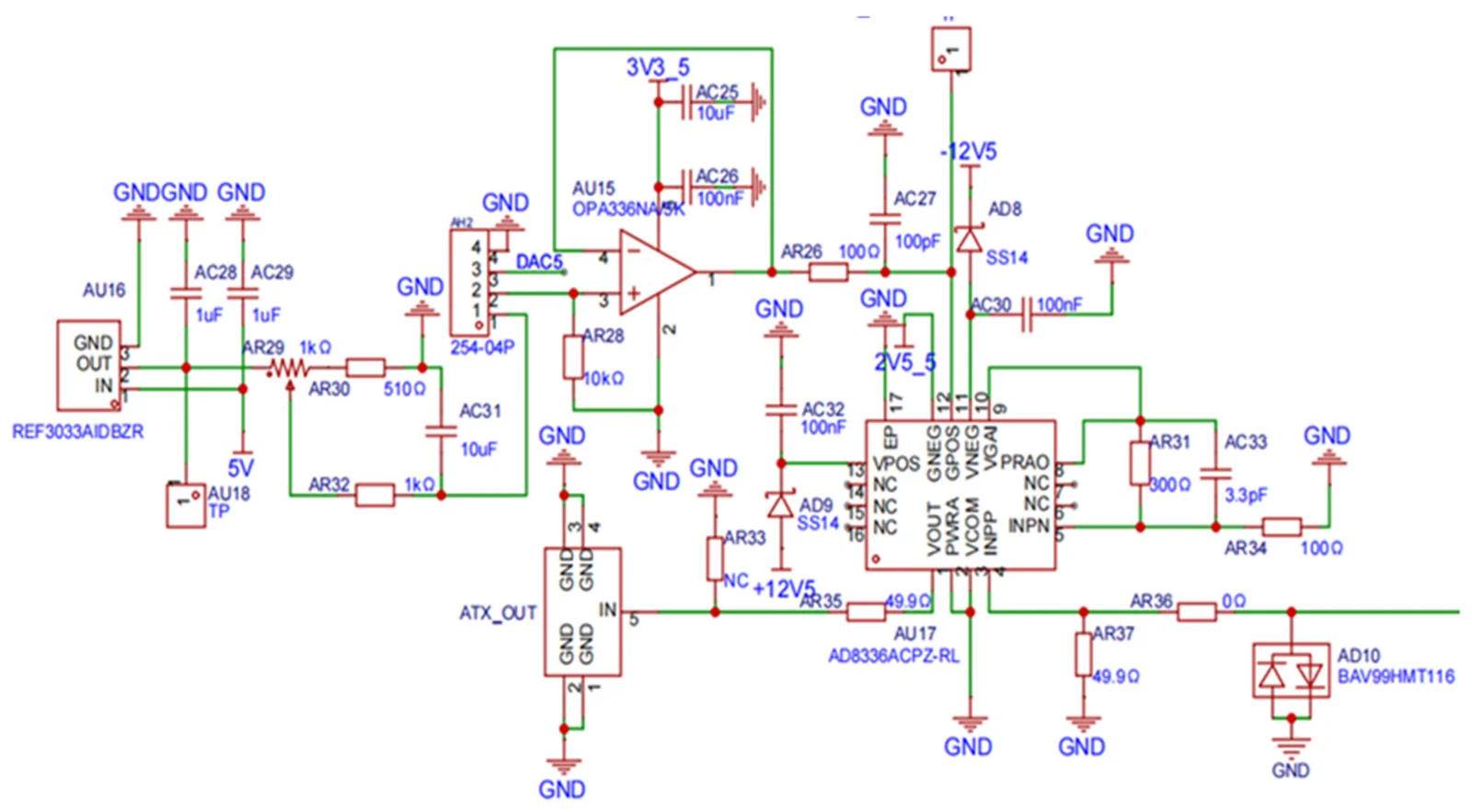
Ultrasonic signal receiving and amplifying circuit.

Ultrasonic receiving circuit: By limiting, amplifying, filtering, and other processing of the echo signal, a preliminary recognizable simulated echo signal can be obtained. The ultrasonic echo signal is only at the millivolt level and requires amplification of the simulated small signal voltage. Therefore, a two-stage amplification circuit was designed to amplify it. The gain of the first stage amplification circuit was fixed, and the gain of the second stage amplification circuit was adjusted according to the actual needs. The AD8336 chip was selected for this system, which has a wide bandwidth to reduce signal distortion after amplification, contain low input noise, and gain programmable adjustment. It is equipped with a non-dedicated preamplifier with a usable gain range of 6 dB to 26 dB. The specific design circuit diagram is shown in Figure 11.

**Figure 12:**
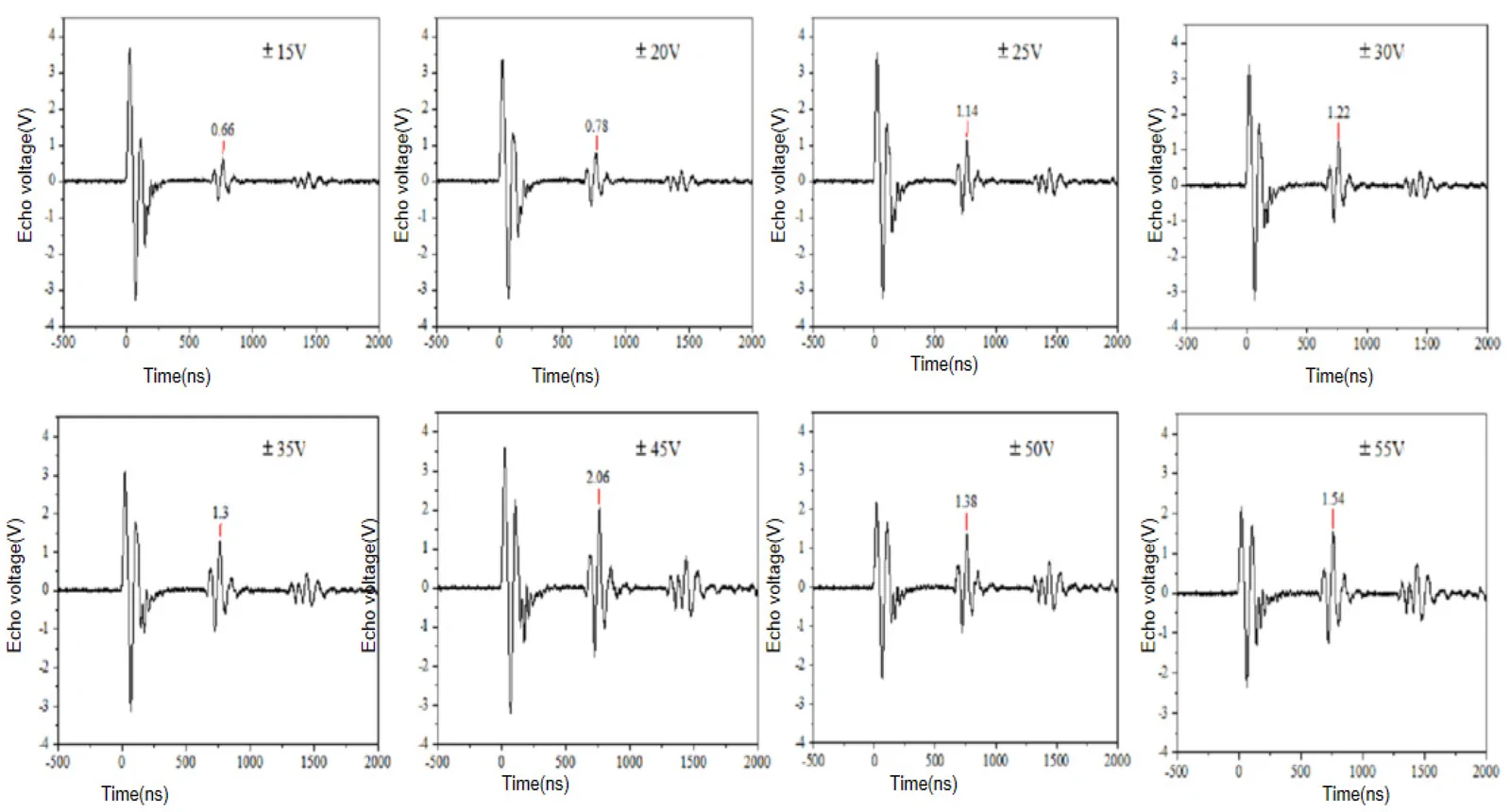
The size of the ultrasonic echo signal input according to different excitation voltages.

To collect and analyze the experimental data, a piezoelectric material ultrasonic testing system software was designed, which can input different excitation frequencies and beam quantities. The echo signal was designed to be received by a signal receiving amplifier and transmitted to an oscilloscope for measurement. The sensor performance test proceeded as follows:

Figure 12 shows the amplitude of ultrasonic echo signals under excitation voltages ranging from ±15 V to ±60 V.

**Figure 13:**
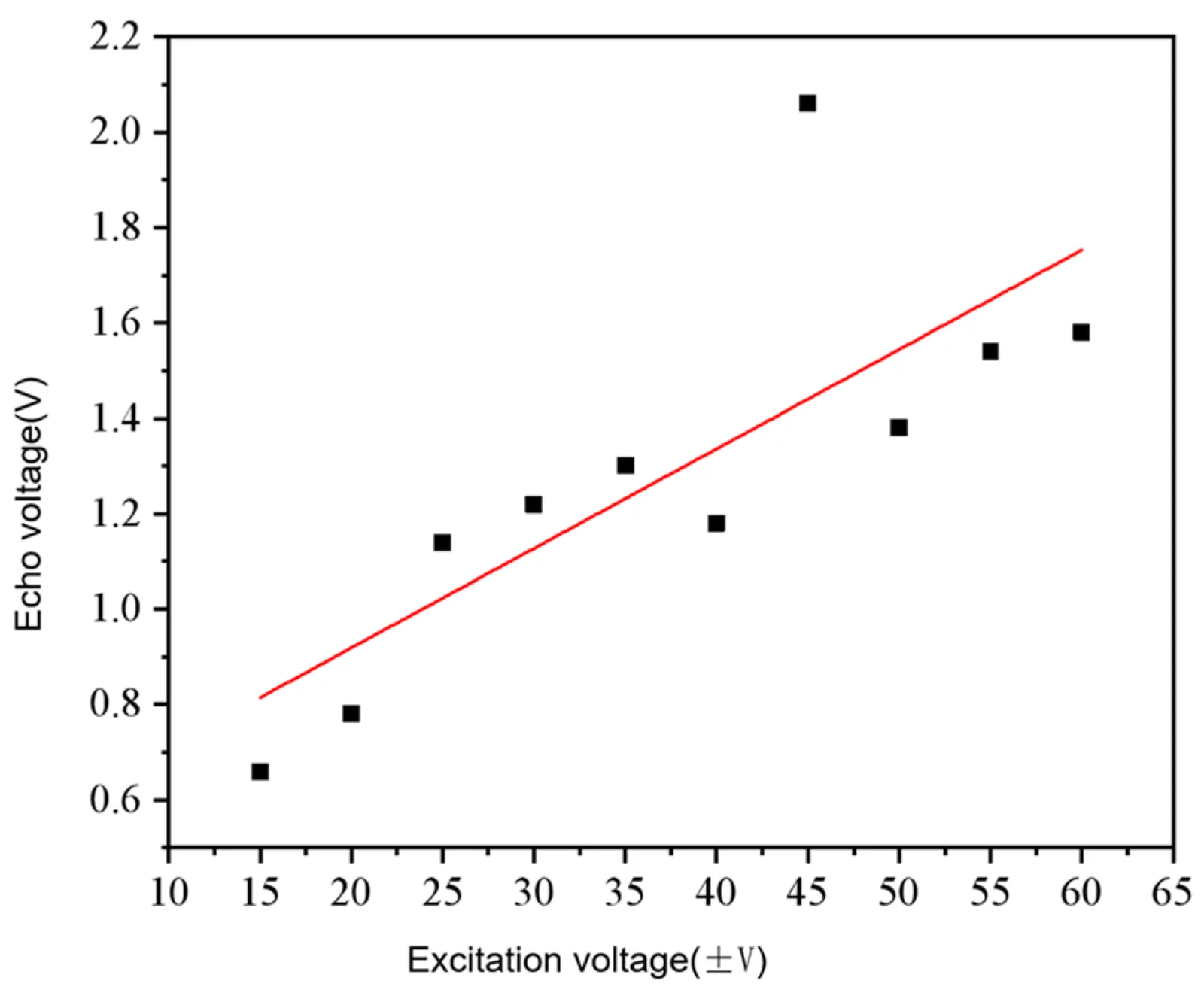
Linear relationship between different excitation voltage inputs.

In order to more intuitively observe the size relationship between the ultrasonic excitation signal and echo signal, the data relationship obtained from the test was fitted. The excitation voltage was between ±15 V and ±60 V, Figure 13 shows that the amplitude of the ultrasonic echo signal is linearly proportional to the excitation signal. In practical application, the size of the ultrasonic echo signal can be increased by adjusting the power supply until it is convenient for observation.

**Figure 14:**
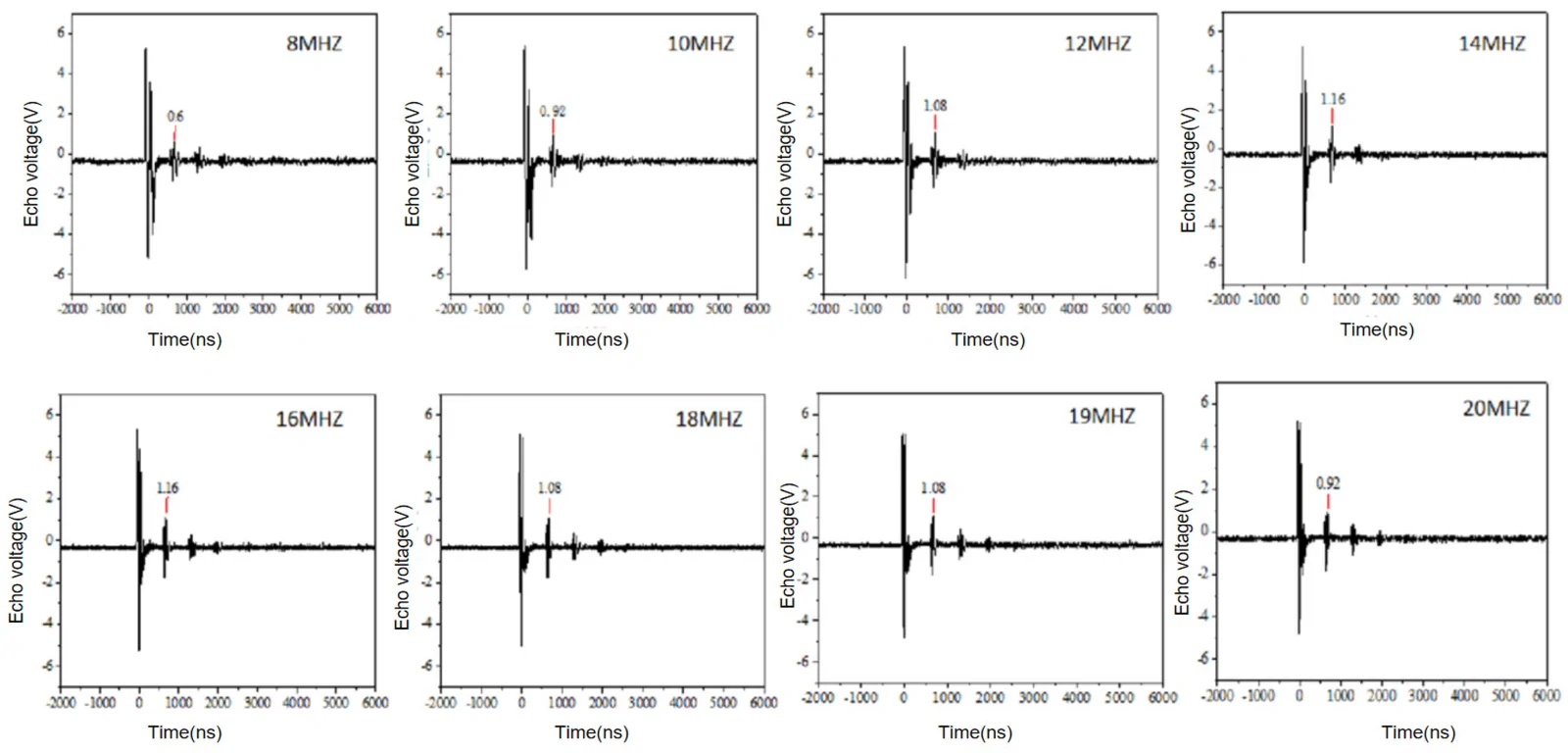
Echo data of different frequency test sensors.

To test the influence of the frequency of different excitation signals on the ultrasonic echo signal, we set the output excitation ultrasonic signal voltage to ±30 V and the ultrasonic output to different frequencies (1 MHz~20 MHz) and determined the corresponding voltage value of the echo. The specific test results are shown in Figure 14.

**Figure 15:**
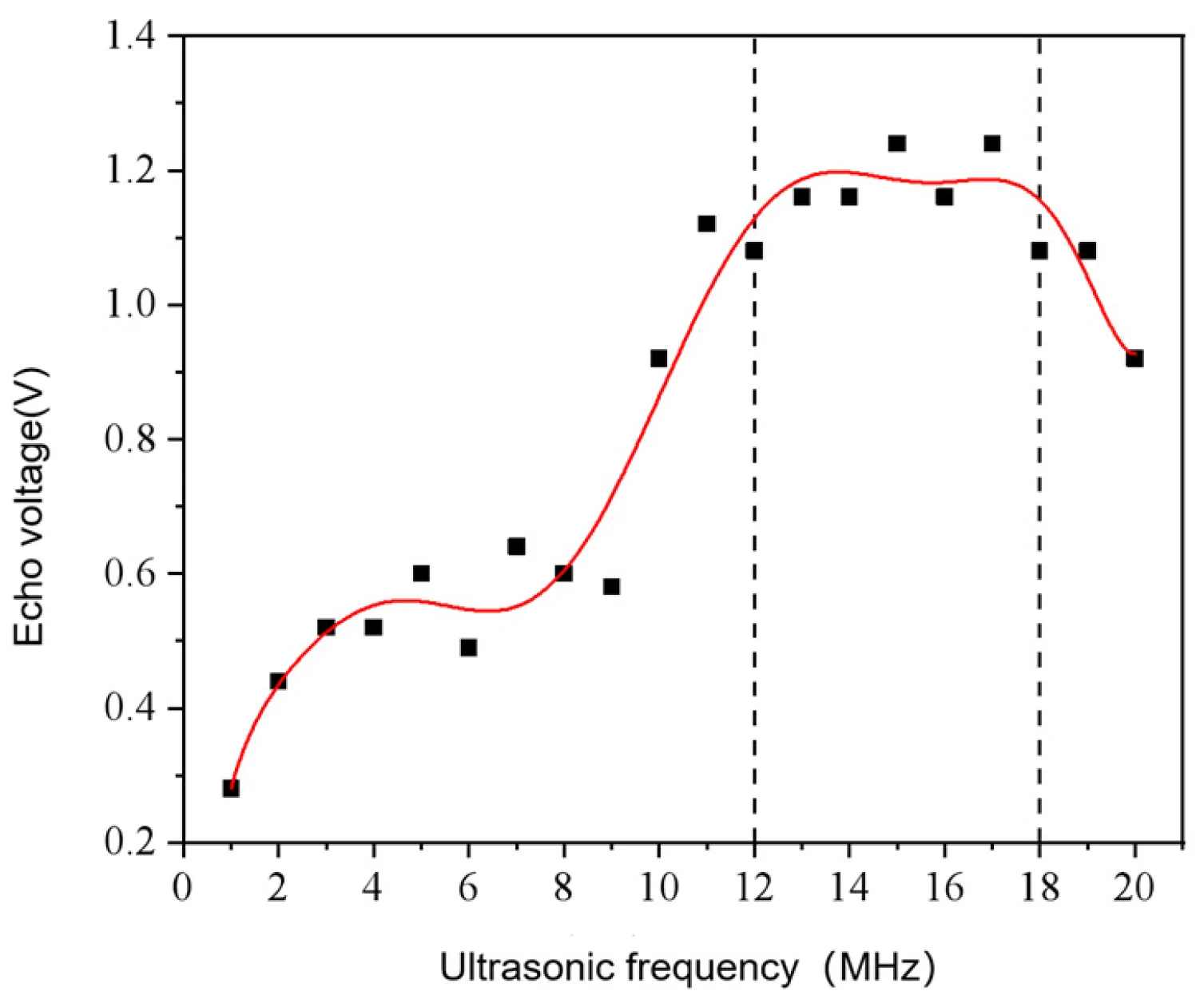
Amplitude frequency curve of the ultrasonic sensor.

At 12 MHz, the echo test waveform signal is the largest, and the output voltage is 1.08 V. Next, 12 MHz~19 MHz retain the maximum value of the signal while, after 20 MHz, the signal becomes smaller and weaker. The amplitude frequency curve of the test sensor is shown in Figure 15, reflecting the signal response strength of the sensor at different ultrasonic frequencies, which is the core spectrum for evaluating the bandwidth, resonance, and sensitivity of ultrasonic transducers [19]. The resonant frequency of the designed sensor is 12 MHz, with a center frequency of −6 dB and an average upper and lower limit frequency of 11.75 MHz. Under the excitation of positive and negative 30 V, the peak amplitude is 1.08 V and the sensitivity meets the requirements of ultrasonic testing.

### 4.3. Ultrasonic Application of Flexible Piezoelectric Sensors

**Figure 16:**
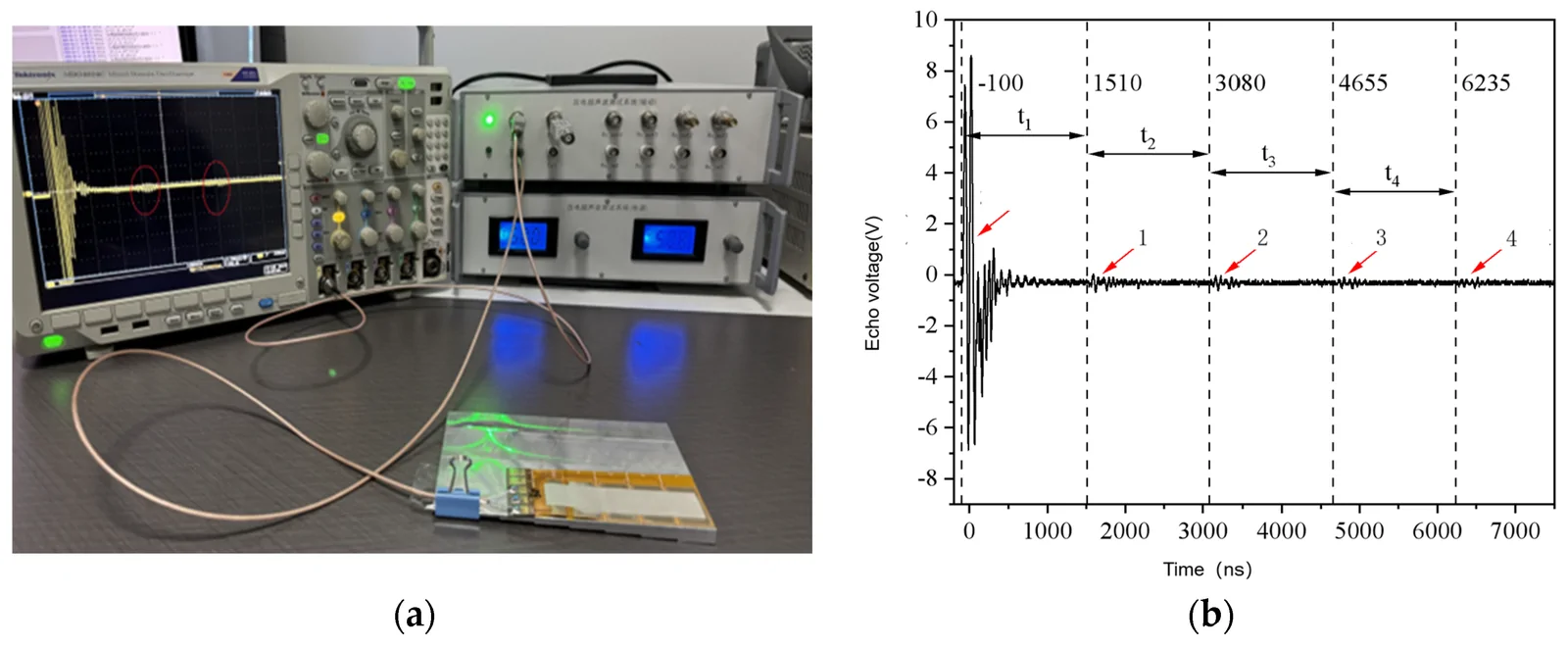
(**a**) Ultrasonic testing of aluminum step block; (**b**) ultrasonic echo test diagram for testing 5 mm aluminum parts (Echo time: t1 = 1610 ns, t2 = 1570 ns, t3 = 1575 ns, t4 = 1580 ns). The red arrows indicate the ultrasonic excitation, primary echo, secondary echo, tertiary echo and quaternary echo.

Ultrasonic non-destructive testing commonly employs four methods based on different application scenarios and testing requirements: pulse reflection, transmission, resonance, and the Lamb wave method [20,21,22]. Here, the PVDF flexible piezoelectric thin-film sensor operates as a bidirectional passive device based on the piezoelectric effect, capable of transmitting and receiving ultrasonic waves. Since the ultrasonic transmitting and receiving probes are located on the same side of the stepped aluminum plate, the pulse reflection method is the most suitable approach for testing, as shown in Figure 16.

The average interval is 1584 ns; thus, the propagation speed of the sound velocity in the aluminum plate can be obtained.

(1) $$V_{AL}=\frac{d_{distance}}{t_{time}}=\frac{5\times 2\times 10^{-3}}{1584\times 10^{-9}}=6313\;m/s$$

The sensor is attached to the thin-walled step block. Four different thicknesses of 5 mm, 6 mm, 7 mm, and 8 mm were selected for the test pieces, corresponding to the four pad receiving pins of the sensor, respectively. The test results of the ultrasonic echo signal diagram are as follows.

According to the principle of ultrasonic thickness measurement, we calculated the thickness of the tested aluminum block as

(2) $${Thick}_{5\mathrm{mm}}=\frac{V_{AL}\times t}{2}=\frac{6313\;m/s\times 1564.4\times 10^{-9}s}{2}\times 10^{-3}=4.938\mathrm{mm}$$

(3) $${Thick}_{6\mathrm{mm}}=\frac{V_{AL}\times t}{2}=\frac{6313\;m/s\times 1886.8\times 10^{-9}s}{2}\times 10^{-3}=5.956\mathrm{mm}$$

(4) $${Thick}_{7\mathrm{mm}}=\frac{V_{AL}\times t}{2}=\frac{6313\;m/s\times 2212.4\times 10^{-9}s}{2}\times 10^{-3}=6.983\mathrm{mm}$$

(5) $${Thick}_{8\mathrm{mm}}=\frac{V_{AL}\times t}{2}=\frac{6313\;m/s\times 2522\times 10^{-9}s}{2}\times 10^{-3}=7.959\mathrm{mm}$$

The test error was less than 0.1 mm, which met the requirements of test accuracy.

### 4.4. Application of Flexible Piezoelectric Sensor in the Ultrasonic Testing Pipeline

**Figure 17:**
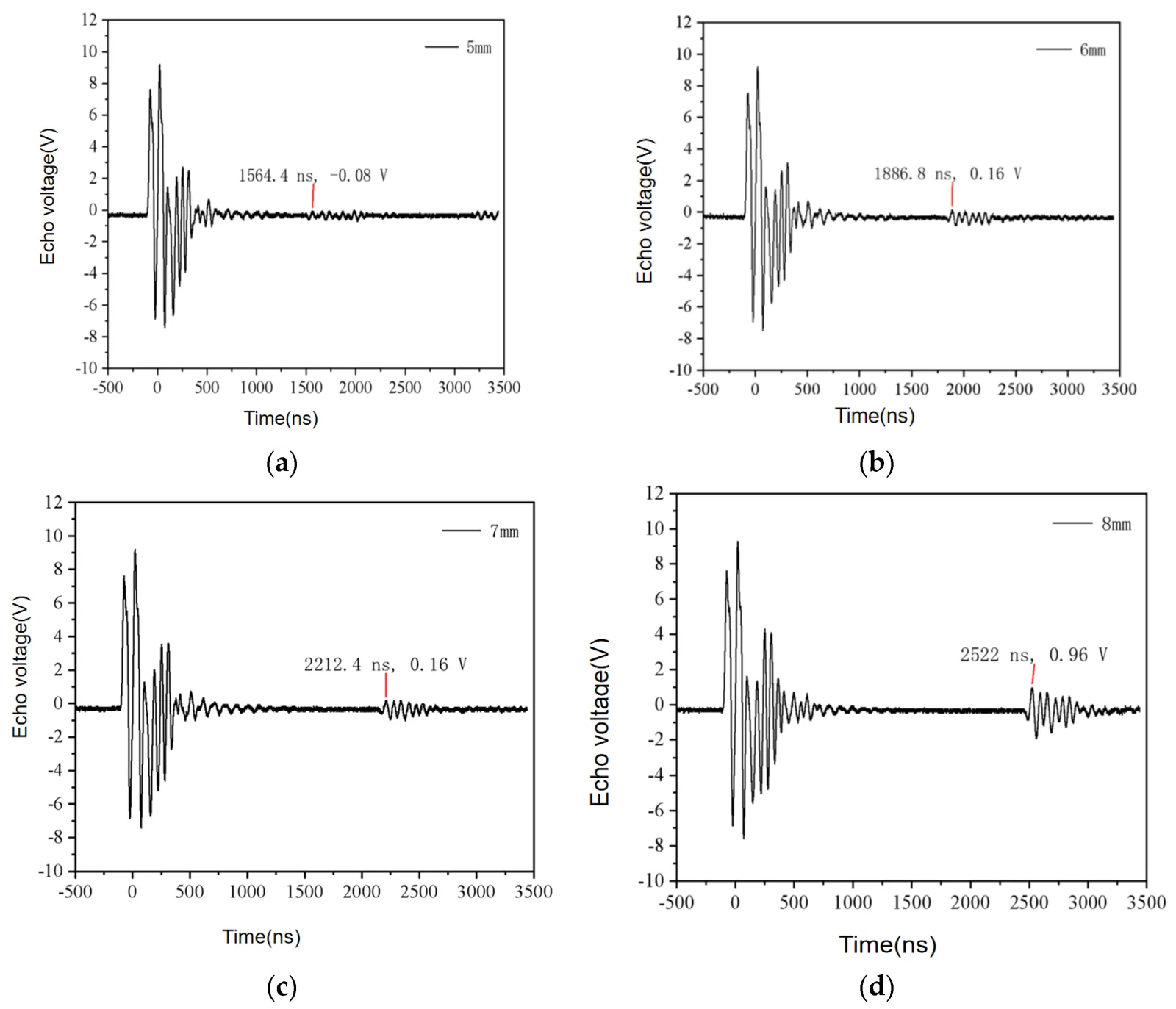
(**a**) Ultrasonic echo test diagram for testing 5 mm aluminum parts (Echo time: T = 1564.4 ns); (**b**) ultrasonic echo test diagram for testing 6 mm aluminum parts (Echo time: T = 1886.8 ns); (**c**) ultrasonic echo test diagram for testing 7 mm aluminum parts (Echo time: T = 2212.4 ns); (**d**) ultrasonic echo test diagram for testing 8 mm aluminum parts (Echo time: T = 2522 ns).

Flexible PVDF ultrasonic sensors can adhere better to surfaces such as pipelines and can be used in the field of pipeline wall thickness monitoring [23]. The tested object in the experiment is the pipeline shown in Figure 17, with a thickness of 10 mm; the sensor selected two test receiver points.

**Figure 18:**
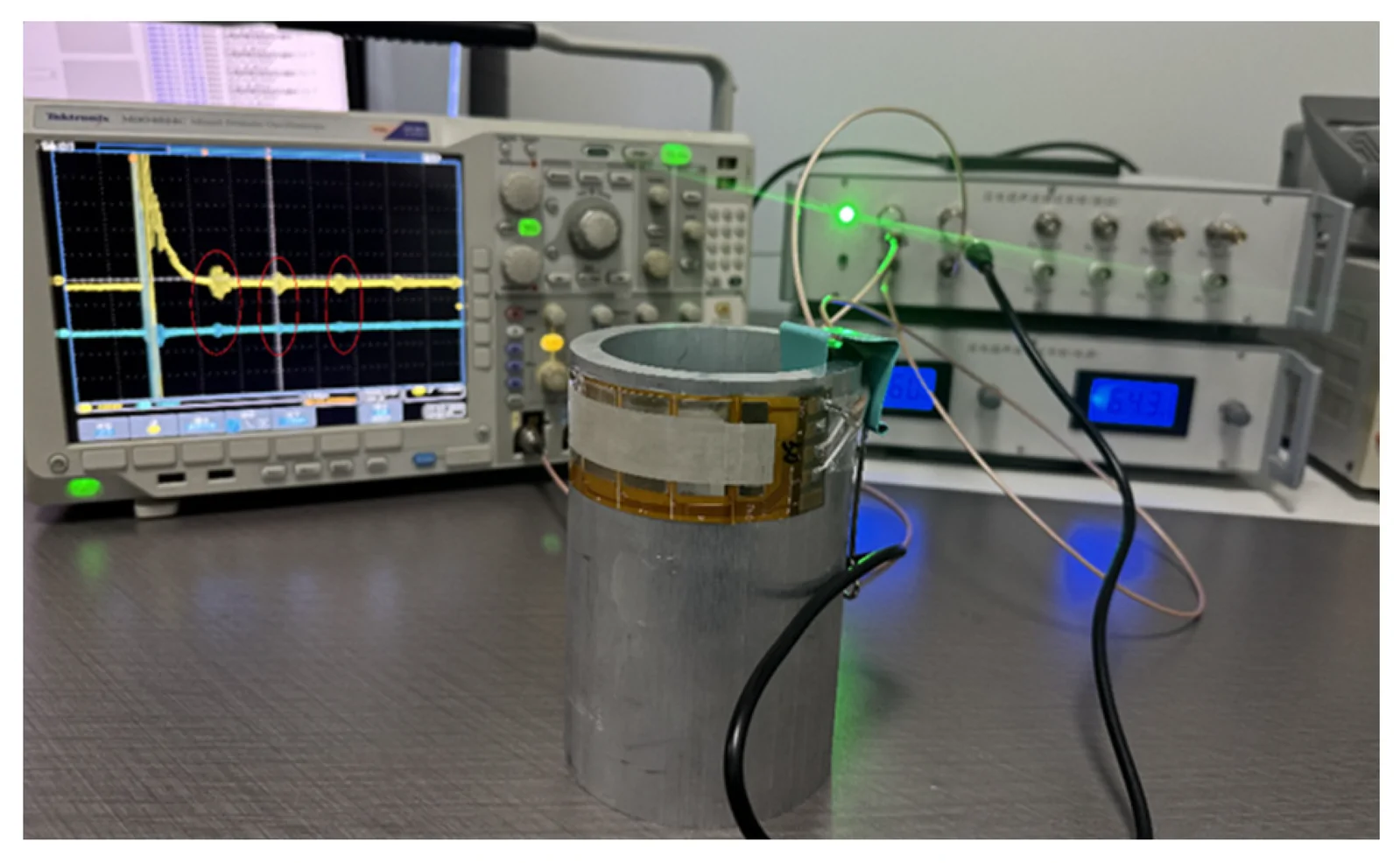
Piezoelectric thin-film sensor testing pipeline.

**Figure 19:**
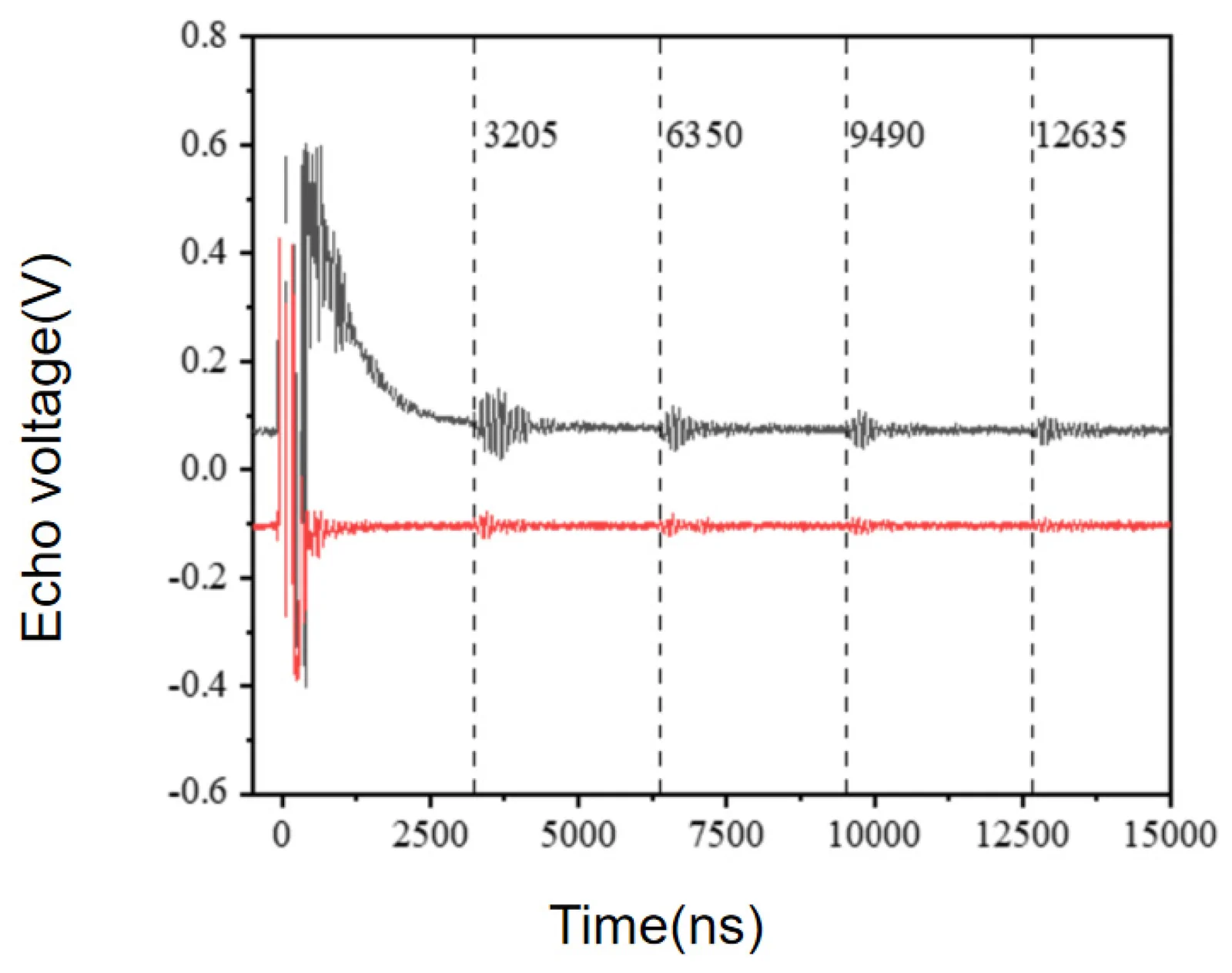
Echo waveform of the ultrasonic thickness measurement for the pipeline.

The sensor monitoring system measured pipeline specimens via ultrasonic echo, as shown in Figure 18, and the ultrasonic echo signals acquired in the pipeline wall-thickness test are shown in Figure 19.

As the echo waveforms of the two test points were basically the same, indicating that the pipe wall thickness is the same; hence, the measurement data of each sensor had good consistency. According to Figure 19, the average time-of-flight of the echo was 3205 ns. The wall thickness of the pipeline was calculated to be 10.10 mm using the ultrasonic thickness-measurement Formula (6) calculated thickness value error remained within 0.1 mm, with good measurement accuracy.

(6) $$Thickness=\frac{V_{AL}\times t}{2}=\frac{6313\;m/s\times 3205\times 10^{-9}s}{2}\times 10^{-3}=10.01\mathrm{mm}$$

## 5. Results

The previous sections elaborated the design of the entire system, which theoretically achieved monitoring of the wall thickness of oil and gas pipelines. In this section, we describe the results from the design and production of a physical prototype based on the theoretical framework, along with the building of an experimental platform, in order to verify its performance in practical applications.

### Accuracy and Repeatability Experiments

**Figure 20:**
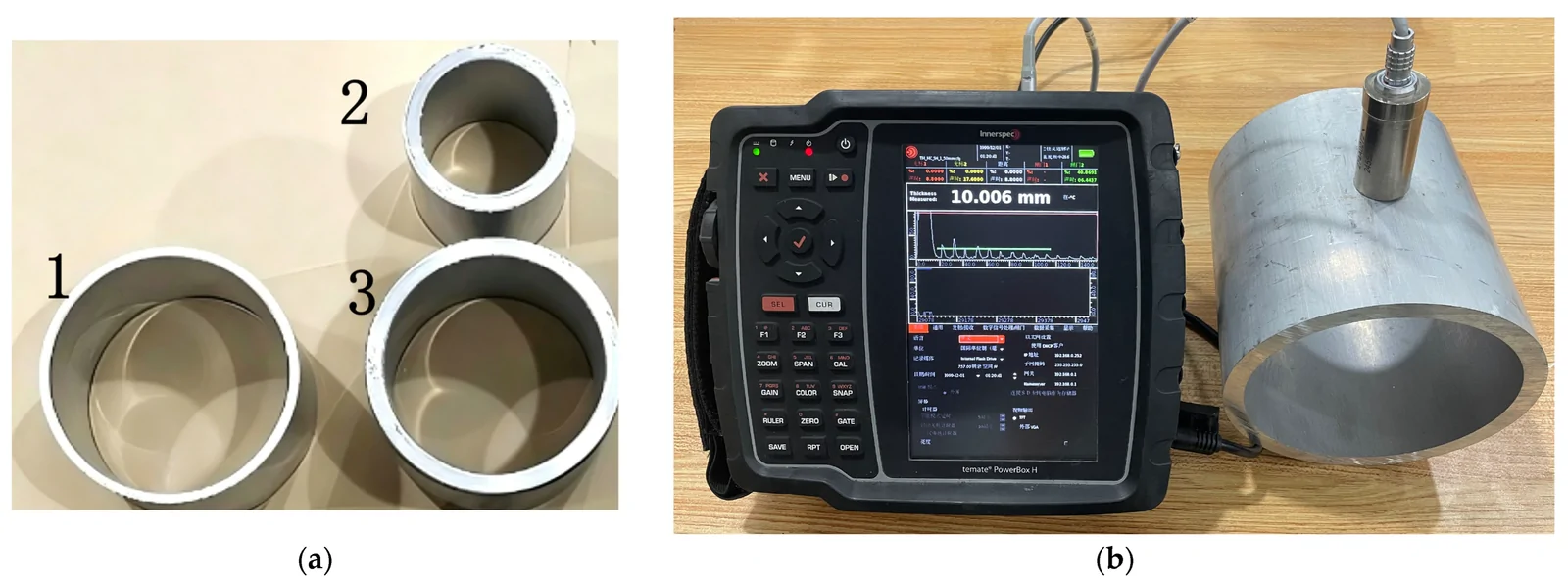
(**a**). Physical image of pipeline specimen; (**b**). pipeline specimen calibration experiment.

The tested specimens were five pipes with different thicknesses and curvatures, as shown in Figure 20. The density of the aluminum pipe specimens was uniform, and the sound velocity at room temperature was 6260 m/s. The specific parameters are shown in Table 4. The wall thickness was calibrated using a portable ultrasonic detector from Innerspec, Forest, VI, USA, as shown in Figure 20 (with a resolution of 0.001 mm and a measurement accuracy of 0.01 mm).

As shown in Table 4, the data obtained by the flexible piezoelectric sensor and processed by the hardware and software in the monitoring system showed that the calculated thickness value error remained within ±0.01 mm, with good measurement accuracy. At the same time, the measurement data of each sensor showed good consistency.

## 6. Discussion

This article focused on the testing application needs of oil pipelines and combined the advantages of ultrasonic non-destructive testing to conduct research on an ultrasonic flexible piezoelectric sensor. The main innovations are detailed as follows.

This work systematically investigated the complete technical chain of flexible piezoelectric thin-film sensors, including piezoelectric material modification, sensor substrate design, sensor fabrication procedures, and the construction of the ultrasonic testing platform. A scalable fabrication route for flexible piezoelectric sensors was proposed, which supports the rapid customization of flexible piezoelectric thin-film sensors for diverse application scenarios. The quantity and size of testing points on the sensor substrate were configured through FPC circuit design. The ZnO doping ratio and fabrication parameters of P(VDF-TrFE) were studied, and the characterizations verified that the piezoelectric performance was significantly improved.

Based on the non-destructive thickness testing requirements for irregularly shaped pipeline components, the problem of the poor contact between traditional block ultrasonic sensors and curved pipelines was solved. The basic shape and testing points of the sensor were designed through an FPC circuit, and the flexible piezoelectric sensor was optimized through custom-doped ZnO/P(VDF-TrFE) material, sensor stack design, and sensor manufacturing process design. The ultrasonic testing system for the sensor was designed to achieve a detection accuracy of 0.01 mm.

Although the ultrasonic thickness measurement system studied in this work achieved its overall design and testing application, there are still many areas for improvement in order to further research and development.

(a) According to the requirements of different working environments, flexible piezoelectric thin-film sensors need to be equipped with customized matching layers and backing layers to further improve their performance. Therefore, further theoretical simulation analysis based on COMSOL Multiphysics 6.4 should be carried out, which can provide guidance for subsequent sensor stack design, fabrication, and performance parameter optimization.
(b) The experiments conducted in this paper were all performed at room temperature. However, in practical applications, the temperature may vary due to the influence of the industrial site environment, thereby affecting the speed of sound. Therefore, further research can be conducted on the impact of temperature on the measurement results, and functions such as temperature correction can be developed to enhance the adaptability of the system to various working environments [24].
(c) Subsequent work will investigate the durability and fatigue performance of the sensor. Long-term dynamic cyclic excitation simulations and experimental characterizations will be implemented to analyze the mechanical fatigue damage and electrical property degradation of the laminated curved sensor. The long-term service stability of the sensor will be explored to offer a sufficient theoretical basis and experimental data for practical engineering deployment.

## Figures and Tables

**Table 1:** Main materials required for the experiment.

Instrument	Specification	Manufacturer
Ultrapure Water System	ACD-0501-U	Yiyang Enterprise Development Co., Ltd. Yiyang, Hunan, China.
Ultrasonic Cleaner	PS-40A	Jiekang Ultrasonic Equipment Co., Ltd., Dongguan, Guangdong, China
Electronic Balance	ATY124	SHIMADZU, Kyoto, Japan
Magnetic Heating Stirrer	08-02	Meiyingpu Instrument & Meter Co., Ltd., Shanghai, China
Forced Air Oven	DHG-9036A	Shanghai Jinghong Experimental Equipment Co., Ltd., Shanghai, China
Plasma Cleaner	Smart plasma 10	OBOE Instruments, Tianjin, China
Numerical Control Film Coater	BEVS1818	Shenghua Industrial Co., Ltd. Changsha, Hunan, China.
Vacuum Drying Oven	/	/
In Situ Polarization Equipment	/	/
Dual-chamber Magnetron Sputtering Deposition System	JGP-560C	Shenyang Scientific Instruments Co., Ltd. Shenyang, Liaoning, China

**Table 2:** The absorption intensities of ZnO/P(VDF-TrFE) at 764.16 cm^−1^ and 839.85 cm^−1^ and the calculated *F_β_* results.

Wave Number	Transmittance (a.u.)					
	PVDF	3 wt% ZnO/PVDF	5 wt% ZnO/PVDF	7 wt% ZnO/PVDF	9 wt% ZnO/PVDF	11 wt% ZnO/PVDF
764.16 cm^−1^	87.2384	87.4077	89.12811	88.06903	89.41881	88.60676
839.85 cm^−1^	28.36578	28.77814	34.47238	31.35699	37.17896	34.40321
*F_β_*	20.51%	20.72%	23.49%	22.03%	24.81%	23.56%

**Table 3:** Basic parameters of thin-film sensors.

Physical Property	Parameter Value
D33	33 pC/N
Dielectric constant	20 (9 wt% ZnO)
PET thickness	50 μm
PI thickness	25 μm
CU pad thickness	35 μm
PVDF thickness	20 μm
Ag thickness	10 μm
Test point length	30 mm
Test point width	16 mm
Test point num	4

**Table 4:** Test results of pipeline thickness using flexible piezoelectric sensors.

Sensor No.	Pipeline 1 (Thickness 5.001 mm)		Pipeline 2 (Thickness 10.007 mm)		Pipeline 3 (Thickness 10.006 mm)	
	Measured (mm)	Error (mm)	Measured (mm)	Error (mm)	Measured (mm)	Error (mm)
1	5.007	0.006	10.008	0.001	9.993	−0.013
2	5.007	0.006	9.990	−0.017	10.020	0.014
3	4.998	−0.003	9.996	−0.011	10.023	0.017
4	4.995	−0.006	10.020	0.013	10.002	−0.004

## Data Availability

The original contributions presented in this study are included in the article. Further inquiries can be directed to the corresponding author.
